# BMP6-Engineered MSCs Induce Vertebral Bone Repair in a Pig Model: A Pilot Study

**DOI:** 10.1155/2016/6530624

**Published:** 2015-12-07

**Authors:** Gadi Pelled, Dmitriy Sheyn, Wafa Tawackoli, Deuk Soo Jun, Youngdo Koh, Susan Su, Doron Cohn Yakubovich, Ilan Kallai, Ben Antebi, Xiaoyu Da, Zulma Gazit, Hyun Bae, Dan Gazit

**Affiliations:** ^1^Department of Surgery, Cedars-Sinai Medical Center, Los Angeles, CA 90048, USA; ^2^Board of Governors Regenerative Medicine Institute, Cedars-Sinai Medical Center, Los Angeles, CA 90048, USA; ^3^Biomedical Imaging Research Institute, Cedars-Sinai Medical Center, Los Angeles, CA 90048, USA; ^4^Skeletal Biotech Laboratory, Hadassah Faculty of Dental Medicine, Hebrew University, Jerusalem, Israel; ^5^Department of Orthopaedic Surgery, Gil Medical Center, Gachon University of Medicine & Science, Incheon, Republic of Korea; ^6^Department of Orthopedics, School of Medicine, Ewha Womans University, Seoul, Republic of Korea; ^7^US Army Institute of Surgical Research, Fort Sam Houston, San Antonio, TX 78234, USA

## Abstract

Osteoporotic patients, incapacitated due to vertebral compression fractures (VCF), suffer grave financial and clinical burden. Current clinical treatments focus on symptoms' management but do not combat the issue at the source. In this pilot study, allogeneic, porcine mesenchymal stem cells, overexpressing the BMP6 gene (MSC-BMP6), were suspended in fibrin gel and implanted into a vertebral defect to investigate their effect on bone regeneration in a clinically relevant, large animal pig model. To check the effect of the BMP6-modified cells on bone regeneration, a fibrin gel only construct was used for comparison. Bone healing was evaluated in vivo at 6 and 12 weeks and ex vivo at 6 months. In vivo CT showed bone regeneration within 6 weeks of implantation in the MSC-BMP6 group while only minor bone formation was seen in the defect site of the control group. After 6 months, ex vivo analysis demonstrated enhanced bone regeneration in the BMP6-MSC group, as compared to control. This preclinical study presents an innovative, potentially minimally invasive, technique that can be used to induce bone regeneration using allogeneic gene modified MSCs and therefore revolutionize current treatment of challenging conditions, such as osteoporosis-related VCFs.

## 1. Introduction

Osteoporosis is a debilitating systemic disorder with severe clinical and financial implications. It is estimated that osteoporosis affects 44 million Americans and more than 200 million people worldwide [1, 2]. In the United States alone, the direct annual cost of osteoporotic fractures was 19 billion dollars in 2005 and with the increase in life expectancy it is predicted to surge to an estimated $60 billion by 2030 [3–5]. Osteoporotic vertebral compression fractures (OVCFs) are the most common type of fractures of osteoporotic patients [6]. It is estimated that, in the United States alone, 1.5 million people suffer from OVCFs every year [7]. Current treatments aim at preventing OVCFs in osteoporotic patients by inhibiting bone resorption using various drugs. These drugs, however, are only efficient for a limited duration and are also associated with severe side effects [8–11]. Once OVCF occurs, nonsurgical treatments fail to treat the source or eliminate the pain and oftentimes lead to physical disability. The limitations of nonoperative management have fostered an increasing interest in new, minimally invasive, surgical approaches. Still, minimally invasive techniques, such as vertebroplasty and kyphoplasty, are associated with a number of serious clinical complications, such as infection, leakage into the spinal canal, and increased incidence of fracture to adjacent vertebrae [12–14]. Furthermore, in two recent clinical studies surgical intervention using PMMA injection was not more effective than a sham treatment [15, 16]. For these reasons, there is a clear medical need to develop new treatment options to combat VCFs in osteoporotic patients.

During the last decade stem cell-based therapies have gained considerable clinical attention due to their potential promise to treat a wide range of disorders, as shown in animal models. The goal of stem cell therapy for osteoporosis is to reduce the susceptibility of fractures by facilitating new bone formation at osteoporotic sites [17]. Mesenchymal stem cells (MSCs) have been shown to induce bone formation and fracture repair in numerous animal studies [18]. We have previously shown that bone morphogenetic protein- (BMP-) modified adipose tissue-derived stem cells induce vertebral defect regeneration in rodents [19]. BMP-engineered MSCs not only secrete BMP to promote osteogenesis but also differentiate into bone-forming cells in response to osteogenic induction [20]. We have also previously shown that MSCs overexpressing BMP6 are more potent in bone formation than MSCs overexpressing BMP2 both in vitro and in vivo [21].

In this pilot study, we hypothesized that transplantation of allogeneic MSCs overexpressing BMP6 will lead to accelerated, robust, and localized bone formation, which could be an attractive therapy for a variety of conditions involving bone loss. In order to test this hypothesis, MSCs were isolated from the bone marrow of donor animals and subsequently transfected via nonviral technique (nucleofection) to overexpress the BMP6 protein. These cells were then suspended in fibrin gel and transplanted into a vertebral defect of a porcine model. For comparison, a fibrin gel only construct was used as control. Bone regeneration was examined in vivo via clinical CT at 6 and 12 weeks and ex vivo via *μ*CT and histology at 6 months.

## 2. Materials and Methods

### 2.1. Cell Preparation

#### 2.1.1. Isolation of BM-Derived MSCs

Allogeneic porcine MSCs were isolated from the bone marrow (BM) of donor Yucatan minipigs as previously described [22]. Briefly, costal BM of euthanized minipigs (35–40 kg with a mean age of 1.5 years) was harvested aseptically. The BM-containing ribs were scraped, flushed with PBS, and centrifuged at 900 g for 10 min. The pellet was resuspended in PBS, after which it was layered on lymphocyte separation medium (ICN Pharmaceuticals, Bryan, OH, USA). Following cellular isolation, the mononuclear cells were plated at a density of 0.4 × 10^6^ cells per cm^2^ and culture medium was changed every 3-4 days. Upon confluence, the cells were replated at a density of 7 × 10^4^ cells per cm^2^ for expansion. For this study, up to fifth passage cells were used for in vivo implantation.

#### 2.1.2. Genetic Modification of MSCs

Genetic modification of porcine MSCs was performed with the aid of a Nucleofector device (Amaxa Biosystems, Cologne, Germany), as previously described [21, 23, 24]. Briefly, 2 × 10^6^ MSCs were transfected with 10 mg of cDNA3-pCMV-rhBMP-6 (rhBMP-6) plasmid. Immediately after transfection, the cells were placed in complete growth medium (including 20% fetal calf serum) and maintained in culture for 24 hrs.

To estimate the amount of rhBMP-6 that was secreted after nucleofection, MSCs from three different donors were grown in confluent conditions in culture for 14 days. Media from the flasks were collected on Day 2 after nucleofection. To quantify the rhBMP-6 secreted during 24 hours, the media were changed 24 hours before sampling. An enzyme-linked immunosorbent assay (ELISA) (R&D Systems, Minneapolis, MN, USA) was performed to measure the amount of rhBMP-6 protein secreted into the culture media by the nucleofected porcine MSCs and compared to the cells nucleofected with GFP reporter gene. After sampling the media, we lifted and counted cells so that we could normalize the secreted protein to 10^6^ cells.

### 2.2. Vertebral Defect Model

The Institutional Animal Care and Use Committee of Cedars-Sinai Medical Center approved all animal procedures used in this study. Six minipigs were used to generate vertebral defects. In each pig, one vertebral defect was generated. Three pigs were treated with fibrin gel (FG) alone (Tisseel, Baxter, IL, USA), and three were treated with BMP6-MSCs (4 × 10^6^) suspended in fibrin gel.

#### 2.2.1. Pre-Op Care and Surgical Anesthesia

Following an 18-hour preoperative fast, each pig was sedated with intramuscular drugs (acepromazine 0.25 mg/kg, ketamine 20 mg/kg, and atropine 0.02–0.05 mg/kg), following which the animal was injected intravenously with propofol (2 mg/kg) to induce full anesthesia. After this had been achieved, the trachea was intubated and anesthesia was maintained using 1–3.5% isoflurane inhaled via the tracheal tube for the duration of the procedure.

*Surgical Procedure*. A 20-cm posterolateral skin incision was made over the lumber region (L1–5), which was then exposed by a lateral transpsoas retroperitoneal approach. One critical-size cylindrical bone defect, 15-mm in depth and 4-mm in diameter, was created in one lumbar vertebra using a surgical drill bit (4.0 JAC 100 SC, Veterinary Orthopedic Implants, St. Augustine, FL). After surgery, the subcutaneous tissue was closed with an absorbable subcutaneous suture and the skin with an absorbable subcuticular suture.

The cDNA3-pCMV-rhBMP-6 nucleofected cells were lifted, counted, and divided to aliquots. Four million cells were suspended in 400 *μ*L fibrin gel (FG; Tisseel kit, Baxter, IL, USA) and then implanted in each vertebral defect. As a control three vertebral defects were treated with 400 *μ*L fibrin gel only (FG only). Next, the subcutaneous tissue layer was closed in a continuous pattern using an absorbable/coated suture and the skin was closed in a subcuticular pattern using a nonabsorbable interrupted over-and-over suture, which was removed 2 weeks after surgery. Finally, the skin area was cleansed with sterile gauzes and 0.5% chlorhexidine gluconate.

#### 2.2.2. Post-Op Care

Buprenorphine (0.1 mg/kg) was administered IM for pain relief immediately after the surgery and every 12 hours during the first day (if needed). Body temperature, pulse, and respiration were closely monitored for potential complications, such as infection.

### 2.3. Evaluation of Bone Regeneration

Bone regeneration was monitored in vivo at 6 and 12 weeks for the duration of the study (i.e., 6 months) using a clinical computed tomography (CT) scanner [Biograph PET-CT, Siemens] to verify that the defect had not spontaneously repaired. After 6 months, the pigs were immobilized with intramuscular ketamine (20 mg/kg) and acepromazine (0.25 mg/kg) and euthanized using a veterinary euthanasia solution (1 cc/10 lbs) injected intravenously into an ear vein. Next, the vertebrae were examined ex vivo using high-resolution microcomputed tomography (*μ*CT) and histological techniques.

#### 2.3.1. Ex Vivo Microcomputed Tomography Analysis

Ex vivo, high-resolution assessment of bone regeneration was performed at the end of the study (i.e., 6 months). For this purpose, the animals were euthanized and their spines were subsequently removed. Bone regeneration at the defect site was evaluated using a preclinical cone-beam *μ*CT imaging system (vivaCT 40; Scanco Medical AG, Brüttisellen, Switzerland). Microtomographic slices were acquired using an X-ray tube with a 55 kVp potential and reconstructed at a voxel size of 35 *μ*m. The *μ*CT analysis used for this study was described in detail by Kallai et al. [25]. Briefly, after locating the defect region, the defect margins were aligned to a standard position and a cylindrical volume of interest (VOI; 5 mm in diameter, 15 mm in length) was defined for a 3D evaluation. A constrained 3D Gaussian filter (*σ* = 0.8 and support = 1) was used to partly suppress the noise in the volumes. The bone tissue was segmented from marrow and soft tissue by using a global thresholding procedure [26]. In addition to visual assessments of the structural images, morphometric indices were determined on the basis of microtomographic datasets by using direct 3D morphometry [27]. Evaluation of regenerated bone tissue was made by a qualitative assessment of bone structure based on 2D cross sections and 3D images and a quantitative assessment of bone structure based on microtomographic datasets created using direct 3D morphometry. The following morphometric indices were determined for newly formed bone in the regeneration sites: (1) volume of mineralized bone tissue [BV, mm^3^] and (2) connectivity density [Conn-Dens, 1/mm^3^], which was derived from the Euler number [28], a topologic measure used to describe the porosity of the bone sample and to show the extent of branching in the bone structure.

#### 2.3.2. Histological Analysis

Histological evaluation of bone formation was performed on operated vertebrae after 6 months of study, as previously described [24, 29]. Briefly, the vertebrae were fixed in 4% formalin, decalcified with 0.5 M EDTA in saline (pH 7.4), and embedded in paraffin. Tissue sections were cut at a thickness of 5 microns and subsequently stained using haematoxylin and eosin (H&E) and Masson's Trichrome (MTC) stain.

### 2.4. Statistical Analysis

The vertebral defect model was created in 6 minipigs consisting of 3 pigs/vertebral defects per group. Results are presented as means ± standard error of means. Statistical tests for significance were performed using unpaired, one tail, Student's* t*-test (GraphPad Prism, San Diego, CA), and the minimal criterion for significance was determined to be a probability level less than 0.05.

## 3. Results

**Figure 1:**
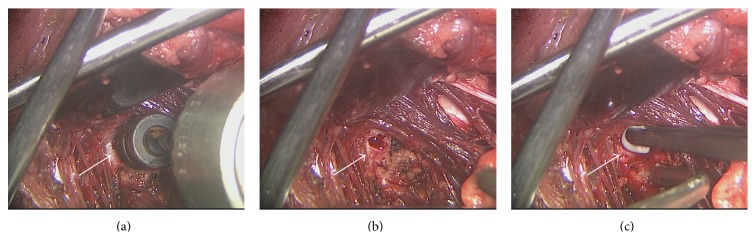
Vertebral bone defect: surgical procedure. Cylindrical bone defects were generated in the lumbar vertebrae of minipigs and treated with either BMP6-MSCs or fibrin gel only. A surgical drill was used to perform the defect in the lumbar vertebral body (a). A 4 mm in diameter, 15-mm deep, cylindrical defect was generated and prepared for construct implantation (b). BMP6-MSCs suspended in fibrin gel or fibrin gel only (control) constructs were implanted in the defect site (c). White arrows point to the location of the defect.

**Figure 2:**
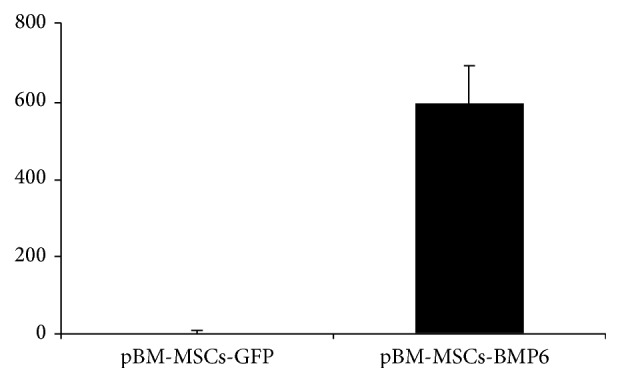
pBM-MSCs nucleofected with rhBMP-6 secrete BMP6 in vitro. Porcine BM-MSCs were nucleofected with cDNA3-pCMV-rhBMP-6 and cultured in vitro. The media were changed after 24 hours and 48 hours after nucleofection the secretion of the BMP6 protein was evaluated using quantitative protein immunoassay (ELISA). The amount of BMP6 secreted was normalized to cell number and compared to the cells that were nucleofected with GFP; bars indicate SE, *n* = 3.

Vertebral defects, 4 mm in diameter and 15 mm in depth, were successfully generated in the vertebral bodies of minipigs (Figure 1). Porcine BM-MSCs were nucleofected with rhBMP-6 encoding plasmid and the secretion of BMP6 protein was verified using an immunoassay (ELISA, Figure 2).

**Figure 3:**
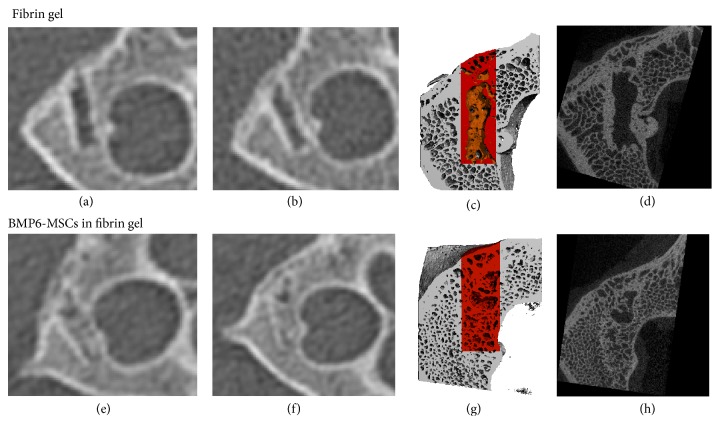
BMP6-MSCs induce vertebral bone repair. Bone regeneration in lumbar vertebral defects was monitored using clinical CT imaging on 6 ((a) and (e)) and 12 weeks after surgery ((b) and (f)). Animals were sacrificed on week 24 (i.e., 6 months) after surgery and excised vertebrae were subjected to *μ*CT imaging. Bone formation was quantified based on *μ*CT scans (analyzed region is highlighted in red, (c) and (g)). Marked differences in bone regeneration can be seen in defects treated with BMP6-MSCs ((g) and (h)) versus fibrin gel only ((c) and (d)).

**Figure 4:**
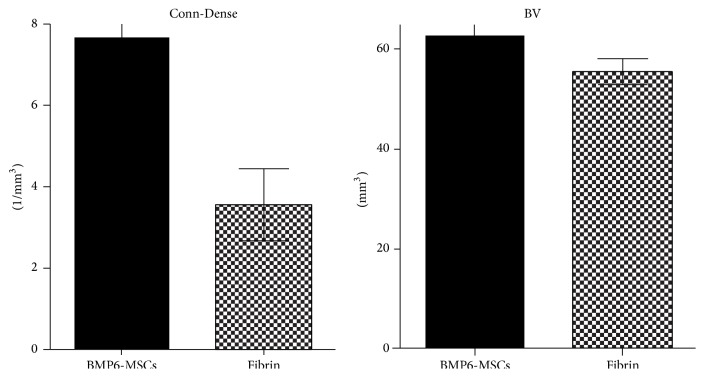
Quantification of bone regeneration in lumbar vertebral defects. Bone volume (BV) and connectivity density (Conn-Dense) parameters were higher in lumbar defects treated with BMP6-MSCs compared to defects treated with fibrin gel only. Statistical significance difference was not reached, probably due to the small sample size (bars indicate SE, *n* = 3).

**Figure 5:**
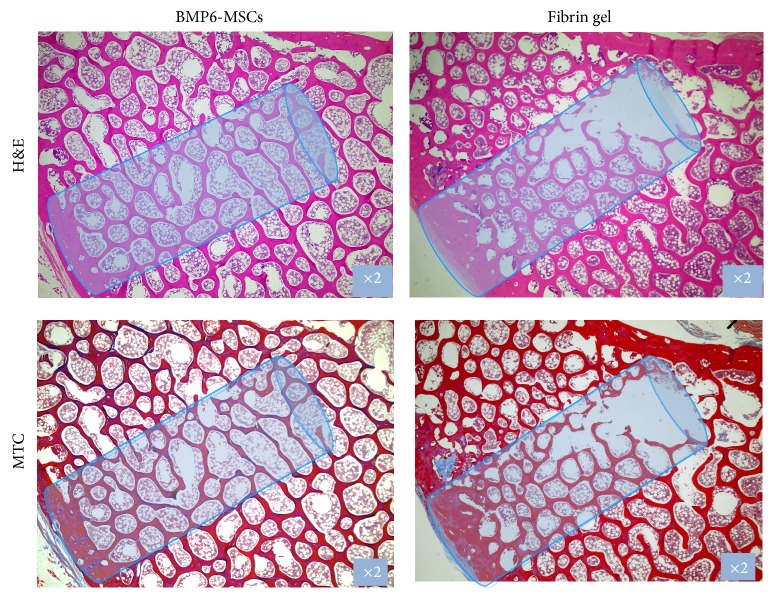
Histological sections of vertebral bone defects. Treated vertebrae were harvested and processed for histology. Sections were stained with H&E and Masson's Trichrome and imaged using a light microscope; representative images showing advanced defect closure in the BMP6-MSC group versus the control (fibrin gel only) group.

Following defect establishment and construct implantation, clinical CT imaging at 6 and 12 weeks revealed considerable repair in the vertebral defect treated with MSCs overexpressing BMP6 compared to those treated with fibrin gel only (Figures 3(a), 3(b), 3(e), and 3(f)). High-resolution *μ*CT analysis of the vertebrae harvested 6 months after surgery showed that the vertebrae architecture was almost completely regenerated in the BMP6-MSCS treated defects. Conversely, the control (fibrin gel only) treated defects less bone repair was visible (Figures 3(c), 3(d), 3(g), and 3(h)). Although significance was not reached, most likely due to the small sample size (*n* = 3), quantitative data derived from the *μ*CT analysis showed higher connectivity density and bone volume indices in defects treated with BMP6-MSCs when compared to control (Figure 4). In addition, histological evaluation of the two treatment groups using H&E and MTC staining further demonstrated the favorable effect of the BMP6-modified MSCs on bone regeneration; that is, after 6 months of study, nearly complete defect closure was evident in the BMP6-MSC group while less bone regeneration was seen in the control group (Figure 5).

## 4. Discussion

The most common type of injury in osteoporotic patients is vertebral compression fracture (VCF). Current surgical, and nonsurgical, interventions for VCFs do not provide appropriate clinical responses to repair and induce new bone formation, frequently leaving patients in pain and/or disability. Bone formation and fracture repair are dependent on the appropriate number and function of resident MSCs. This has inspired investigators to utilize MSCs in a number of preclinical animal models to increase the number of functional MSCs and thereby enhance bone regeneration in vivo [30–40]. Indeed, the field of stem cell therapy has been expanding rapidly over the last two decades. MSCs have also been used in conjunction with gene delivery methods to express various bone inducing factors, such as BMPs, to further augment the bone formation process. Various studies have reported promising results involving genetically modified stem cells as therapeutic vehicles to induce bone regeneration in vivo [18, 19, 21, 29, 41–50]. Our group has previously reported the accelerated repair of a vertebral defect in rats using a local injection of BMP6-modified stem cells [19]. The amounts of BMP6 secreted in the current study were comparable to our previous studies in porcine ASCs [21, 24] and BM-MSCs [21]. Based on these promising results, we hypothesized that BMP6-MSCs administration will accelerate bone formation in a vertebral defect of minipigs. To our knowledge, this is the first study that attempts to promote vertebral bone regeneration via implantation of allogeneic, gene modified stem cells in a large animal model.

To test our hypothesis, a 4 mm in diameter and 15 mm deep cylindrical defect was first created in the lumbar vertebral body of minipigs (Figure 1). Following defect establishment, BMP6-MSCs suspended in fibrin gel, or fibrin gel alone, were implanted into the defect for 6 months. At 6 and 12 weeks after surgery, bone regeneration was evaluated via in vivo clinical CT. Already at 6 weeks, it was shown that the defect treated with the BMP6-MSCs began to heal while the defect treated with fibrin gel alone exhibited less new bone growth (Figure 3). At the study completion (i.e., 6 months), the vertebrae were excised for ex vivo analyses using *μ*CT and histology. Similar to the in vivo CT results, defects treated with the BMP6-modified MSCs were almost entirely repaired whereas those treated with control had less bone growth. Quantitative data obtained from CT scans showed a twofold increase in connectivity density as well as an increase (though not significant) in bone volume in defects treated with the BMP6-MSCs, as compared to control (Figure 3).

One limitation of the study is the small sample size used (*n* = 3). We believe that differences between groups were not significant due to that reason; hence future studies should incorporate more animals to reduce experimental error. Optimization of the technique, such as varying cell loading density and/or type of biomaterial used, may also improve therapeutic outcome. For example, fibrin gel, which has low biomechanical properties, might be replaced by a different biomaterial that may offer an option to increase the vertebral height, while the engineered MSCs induce new bone formation.

An alternative approach might include a systemic treatment. Since MSCs intrinsically home and engraft at sites of injury and inflammation [51, 52], systemic (versus local) delivery of cells may also improve clinical outcome; this is because, unlike local transplantation, cells can migrate toward the wound site in response to physiological cues from the body (such as inflammatory mediators), at the appropriate time and quantity.

One more limitation of the study is the use of healthy animals. Clearly further studies should be conducted in osteoporotic animals. In addition, the feasibility of an image-guided injection of engineered MSCs to the injured vertebra should be demonstrated.

In summary, current treatments for VCFs in osteoporotic patients provide poor patient outcome, an outcome that is not better than sham treatment, which calls for a fresh clinical approach [30]. This pilot preclinical study aims at bridging this gap by presenting a potentially noninvasive technique that delivers BMP6-modified MSCs via local injection to augment bone formation in a large animal model. Our results elude that this technique may be an attractive alternative to substitute or supplement current therapeutic approaches, particularly because the gene delivery method used is nonviral and considered clinically safe [23, 53]. Still, more preclinical studies are needed in order to verify our results and translate this research method into clinical practice.

## References

[1] Cooper C., Campion G., Melton L. J. (1992). Hip fractures in the elderly: a world-wide projection. *Osteoporosis International*.

[2] National Osteoporosis Foundation (2013). *Debunking the Myths*.

[3] Lien C.-Y., Ho K. C.-Y., Lee O. K., Blunn G. W., Su Y. (2009). Restoration of bone mass and strength in glucocorticoid-treated mice by systemic transplantation of CXCR4 and cbfa-1 co-expressing mesenchymal stem cells. *Journal of Bone and Mineral Research*.

[4] Martin K. E., Yu J., Campbell H. E., Abarca J., White T. J. (2011). Analysis of the comparative effectiveness of 3 oral bisphosphonates in a large managed care organization: adherence, fracture rates, and all-cause cost. *Journal of Managed Care Pharmacy*.

[5] Ray N. F., Chan J. K., Thamer M., Melton L. J. (1997). Medical expenditures for the treatment of osteoporotic fractures in the United States in 1995: report from the National Osteoporosis Foundation. *Journal of Bone and Mineral Research*.

[6] Kim D. H., Vaccaro A. R. (2006). Osteoporotic compression fractures of the spine; current options and considerations for treatment. *Spine Journal*.

[7] Alexandru D., So W. (2012). Evaluation and management of vertebral compression fractures. *The Permanente Journal*.

[8] Khan A. A., Sándor G. K. B., Dore E. (2009). Bisphosphonate associated osteonecrosis of the jaw. *Journal of Rheumatology*.

[9] Lo J. C., O'Ryan F. S., Gordon N. P. (2010). Prevalence of osteonecrosis of the jaw in patients with oral bisphosphonate exposure. *Journal of Oral and Maxillofacial Surgery*.

[10] Nieves J. W., Cosman F. (2010). Atypical subtrochanteric and femoral shaft fractures and possible association with bisphosphonates. *Current Osteoporosis Reports*.

[11] Saleh A., Hegde V. V., Potty A. G., Lane J. M. (2013). Bisphosphonate therapy and atypical fractures. *Orthopedic Clinics of North America*.

[12] Frankel B. M., Monroe T., Wang C. (2007). Percutaneous vertebral augmentation: an elevation in adjacent-level fracture risk in kyphoplasty as compared with vertebroplasty. *Spine Journal*.

[13] Lee H. M., Park S. Y., Lee S. H., Suh S. W., Hong J. Y. (2012). Comparative analysis of clinical outcomes in patients with osteoporotic vertebral compression fractures (OVCFs): conservative treatment versus balloon kyphoplasty. *Spine Journal*.

[14] Shen M. S., Kim Y. H. (2006). Vertebroplasty and kyphoplasty: treatment techniques for managing osteoporotic vertebral compression fractures. *Bulletin of the NYU Hospital for Joint Diseases*.

[15] Buchbinder R., Osborne R. H., Ebeling P. R. (2009). A randomized trial of vertebroplasty for painful osteoporotic vertebral fractures. *The New England Journal of Medicine*.

[16] Kallmes D. F., Comstock B. A., Heagerty P. J. (2009). A randomized trial of vertebroplasty for osteoporotic spinal fractures. *The New England Journal of Medicine*.

[17] Liu Y., Wu J., Zhu Y., Han J. (2014). Therapeutic application of mesenchymal stem cells in bone and joint diseases. *Clinical and Experimental Medicine*.

[18] Kimelman N., Pelled G., Helm G. A., Huard J., Schwarz E. M., Gazit D. (2007). Review: gene- and stem cell-based therapeutics for bone regeneration and repair. *Tissue Engineering*.

[19] Sheyn D., Kallai I., Tawackoli W. (2011). Gene-modified adult stem cells regenerate vertebral bone defect in a rat model. *Molecular Pharmaceutics*.

[20] Moutsatsos I. K., Turgeman G., Zhou S. (2001). Exogenously regulated stem cell-mediated gene therapy for bone regeneration. *Molecular Therapy*.

[21] Mizrahi O., Sheyn D., Tawackoli W. (2013). BMP-6 is more efficient in bone formation than BMP-2 when overexpressed in mesenchymal stem cells. *Gene Therapy*.

[22] Bosch P., Pratt S. L., Stice S. L. (2006). Isolation, characterization, gene modification, and nuclear reprogramming of porcine mesenchymal stem cells. *Biology of Reproduction*.

[23] Aslan H., Zilberman Y., Arbeli V. (2006). Nucleofection-based ex vivo nonviral gene delivery to human stem cells as a platform for tissue regeneration. *Tissue Engineering*.

[24] Sheyn D., Pelled G., Zilberman Y. (2008). Nonvirally engineered porcine adipose tissue-derived stem cells: Use in posterior spinal fusion. *Stem Cells*.

[25] Kallai I., Mizrahi O., Tawackoli W., Gazit Z., Pelled G., Gazit D. (2011). Microcomputed tomographyg-based structural analysis of various bone tissue regeneration models. *Nature Protocols*.

[26] Muller R., Ruegsegger P. (1997). Micro-tomographic imaging for the nondestructive evaluation of trabecular bone architecture. *Studies in Health Technology and Informatics*.

[27] Hildebrand T., Laib A., Müller R., Dequeker J., Rüegsegger P. (1999). Direct three-dimensional morphometric analysis of human cancellous bone: microstructural data from spine, femur, iliac crest, and calcaneus. *Journal of Bone and Mineral Research*.

[28] Odgaard A., Gundersen H. J. G. (1993). Quantification of connectivity in cancellous bone, with special emphasis on 3-D reconstructions. *Bone*.

[29] Turgeman G., Pittman D. D., Müller R. (2001). Engineered human mesenchymal stem cells: a novel platform for skeletal cell mediated gene therapy. *Journal of Gene Medicine*.

[30] Antebi B., Pelled G., Gazit D. (2014). Stem cell therapy for osteoporosis. *Current Osteoporosis Reports*.

[31] Florczyk S. J., Leung M., Li Z., Huang J. I., Hopper R. A., Zhang M. (2013). Evaluation of three-dimensional porous chitosan-alginate scaffolds in rat calvarial defects for bone regeneration applications. *Journal of Biomedical Materials Research A*.

[32] Lee G.-S., Park J.-H., Shin U. S., Kim H.-W. (2011). Direct deposited porous scaffolds of calcium phosphate cement with alginate for drug delivery and bone tissue engineering. *Acta Biomaterialia*.

[33] Liu H., Peng H., Wu Y. (2013). The promotion of bone regeneration by nanofibrous hydroxyapatite/chitosan scaffolds by effects on integrin-BMP/Smad signaling pathway in BMSCs. *Biomaterials*.

[34] Liu Y., Ming L., Luo H. (2013). Integration of a calcined bovine bone and BMSC-sheet 3D scaffold and the promotion of bone regeneration in large defects. *Biomaterials*.

[35] Ocarino N. D. M., Boeloni J. N., Jorgetti V., Gomes D. A., Goes A. M., Serakides R. (2010). Intra-bone marrow injection of mesenchymal stem cells improves the femur bone mass of osteoporotic female rats. *Connective Tissue Research*.

[36] Peng H., Wright V., Usas A. (2002). Synergistic enhancement of bone formation and healing by stem cell-expressed VEGF and bone morphogenetic protein-4. *Journal of Clinical Investigation*.

[37] Schubert T., Lafont S., Beaurin G. (2013). Critical size bone defect reconstruction by an autologous 3D osteogenic-like tissue derived from differentiated adipose MSCs. *Biomaterials*.

[38] Terella A., Mariner P., Brown N., Anseth K., Streubel S.-O. (2010). Repair of a calvarial defect with biofactor and stem cell-embedded polyethylene glycol scaffold. *Archives of Facial Plastic Surgery*.

[39] Wang Z., Goh J., Das De S. (2006). Efficacy of bone marrow-derived stem cells in strengthening osteoporotic bone in a rabbit model. *Tissue Engineering*.

[40] Yang C., Frei H., Rossi F. M., Burt H. M. (2009). The differential in vitro and in vivo responses of bone marrow stromal cells on novel porous gelatin-alginate scaffolds. *Journal of Tissue Engineering and Regenerative Medicine*.

[41] Fischer J., Kolk A., Wolfart S. (2011). Future of local bone regeneration—protein versus gene therapy. *Journal of Cranio-Maxillofacial Surgery*.

[42] Jabbarzadeh E., Starnes T., Khan Y. M. (2008). Induction of angiogenesis in tissue-engineered scaffolds designed for bone repair: a combined gene therapy-cell transplantation approach. *Proceedings of the National Academy of Sciences of the United States of America*.

[43] Kim D., Sun W. C., Sun J. H. (2006). Retrovirus-mediated gene transfer of receptor activator of nuclear factor-*κ*B-Fc prevents bone loss in ovariectomized mice. *Stem Cells*.

[44] Bleich N. K., Kallai I., Lieberman J. R., Schwarz E. M., Pelled G., Gazit D. (2012). Gene therapy approaches to regenerating bone. *Advanced Drug Delivery Reviews*.

[45] Kumar S., Wan C., Ramaswamy G., Clemens T. L., Ponnazhagan S. (2010). Mesenchymal stem cells expressing osteogenic and angiogenic factors synergistically enhance bone formation in a mouse model of segmental bone defect. *Molecular Therapy*.

[46] Li R., Stewart D. J., von Schroeder H. P., Mackinnon E. S., Schemitsch E. H. (2009). Effect of cell-based VEGF gene therapy on healing of a segmental bone defect. *Journal of Orthopaedic Research*.

[47] Pelled G., Ben-Arav A., Hock C. (2010). Direct gene therapy for bone regeneration: gene gelivery, animal models, and outcome measures. *Tissue Engineering—Part B: Reviews*.

[48] Riew K. D., Lou J., Wright N. M., Cheng S.-L., Bae K. T., Avioli L. V. (2003). Thoracoscopic intradiscal spine fusion using a minimally invasive gene-therapy technique. *The Journal of Bone & Joint Surgery—American Volume*.

[49] Steinhardt Y., Aslan H., Regev E. (2008). Maxillofacial-derived stem cells regenerate critical mandibular bone defect. *Tissue Engineering A*.

[50] Tang Y., Tang W., Lin Y. (2008). Combination of bone tissue engineering and BMP-2 gene transfection promotes bone healing in osteoporotic rats. *Cell Biology International*.

[51] Jackson W. M., Nesti L. J., Tuan R. S. (2012). Concise review: clinical translation of wound healing therapies based on mesenchymal stem cells. *Stem Cells Translational Medicine*.

[52] Rustad K. C., Gurtner G. C. (2012). Mesenchymal stem cells home to sites of injury and inflammation. *Advances in Wound Care*.

[53] Lu C.-H., Chang Y.-H., Lin S.-Y., Li K.-C., Hu Y.-C. (2013). Recent progresses in gene delivery-based bone tissue engineering. *Biotechnology Advances*.

